# Patient characteristics, burden and pharmacotherapy of treatment-resistant schizophrenia: results from a survey of 204 US psychiatrists

**DOI:** 10.1186/s12888-019-2318-x

**Published:** 2019-11-14

**Authors:** Christoph U. Correll, Thomas Brevig, Cecilia Brain

**Affiliations:** 1 The Zucker Hillside Hospital, Department of Psychiatry, 75–59 263rd Street, Glen Oaks, New York 11004 USA; 2 Department of Psychiatry and Molecular Medicine, The Donald and Barbara Zucker School of Medicine at Hofstra/Northwell, Hempstead, NY USA; 3 Department of Child and Adolescent Psychiatry, Charité-Universitätsmedizin Berlin, corporate member of Freie Universität Berlin, Humboldt-Universität zu Berlin, and Berlin Institute of Health, Berlin, Germany; 4 H. Lundbeck A/S, Valby, Copenhagen, Denmark

**Keywords:** Antipsychotics, Clozapine, Demography, Treatment resistance, Hallucinations, Humans, Prognosis, Psychiatry, Schizophrenia, Surveys and questionnaires

## Abstract

**Background:**

Minimal/non-response to antipsychotic treatment, and persistent positive symptoms despite treatment, are common among patients with schizophrenia. The aim of this study was to characterize a US treatment-resistant schizophrenia (TRS) population in terms of patient demographics, burden of symptoms, treatment history, and factors influencing therapeutic choice.

**Methods:**

In an online survey, 204 psychiatrists self-selected and completed three patient records: two TRS and one schizophrenia (‘non-TRS’).

**Results:**

Respondents reported that 29.5% of their schizophrenia caseload had TRS. Selected TRS (*n* = 408) vs non-TRS (*n* = 204) patients were more likely to be unemployed (74.5% vs 45.1%, *p* < 0.001), hospitalized at least once (93.4% vs 74.0%, *p* < 0.001), and to have physical/psychiatric comorbidities including obesity (40.2% vs 23.5%, *p* < 0.001) and depression (38.7% vs 25.0%, *p* = 0.001). Psychiatric symptoms were more frequent and severe in TRS, and interfered more with social and functioning domains. Of positive symptoms, eliminating delusions and hallucinations was considered most important to improve a patient’s long-term prognosis. In TRS, clozapine monotherapy was the most common treatment (15.9%), though ranked fifth of ten options to treat TRS. Psychiatrists typically increased the antipsychotic dose or added a second antipsychotic before initiating clozapine or switching antipsychotics. Antipsychotic switches were most commonly due to lack of efficacy (TRS = 71.4% vs non-TRS = 54.3%, *p* < 0.001) and intolerability (34.4% vs 38.4%, *p* = 0.22) with the prior antipsychotic. Persistent hallucinatory behavior was the top symptom leading to treatment switches in TRS (63.9% vs 37.1%, *p* < 0.001).

**Conclusions:**

According to psychiatrists, symptoms have a greater clinical burden on patients with TRS than non-TRS. TRS is commonly managed by antipsychotic dose increases/combinations, with clozapine the fifth preference despite being the only approved TRS medication. New treatments are needed for patients who do not respond to available antipsychotics.

## Background

Schizophrenia is a heterogeneous syndrome with regard to the presentation of symptoms and responsiveness to available antipsychotics [1–4]. Minimal/non-response to antipsychotic treatment, and persistent positive symptoms despite treatment, are common among patients with schizophrenia [2–4]. A review of studies in first-episode psychosis found that response rates of around 50% were typical in the first year of treatment (range: 40–87%) [5]. Risk factors for a poorer response to treatment and lower likelihood of remission include patient variables (such as poor premorbid adjustment), illness variables (such as younger age at illness onset, longer duration of untreated psychosis, and, specifically for remission, more severe baseline psychopathology), and treatment variables (such as nonadherence to antipsychotics) [5].

Treatment-resistant schizophrenia (TRS) is broadly defined in clinical guidelines as an inadequate response in target schizophrenia symptoms (often positive symptoms) following treatment with two or more antipsychotic treatments of adequate dose and duration [2, 6–9]. While definition dependent, many authors believe that the prevalence of treatment resistance among patients with schizophrenia is up to 30% [2, 9–12]. Candidate predictors of TRS, identified in a population-based cohort study of patients with schizophrenia in the Danish national registry, included younger age, living in a less urban area, inpatient status at diagnosis, paranoid subtype, and having made a suicide attempt [13].

TRS has a severe clinical and economic impact on patients, carers, families, and society as a whole [14–16]. Whereas drug costs are relatively low in TRS, hospitalization and total health resource utilization costs are considerably higher than in non-treatment-resistant schizophrenia (‘non-TRS’) [14, 15]. Contributing to high hospitalization costs, the hospitalization rate is twice as high in TRS than non-TRS [14]. Considering the burden on patients, unemployment rates are higher in TRS than non-TRS, and cognitive functioning and global psychosocial functioning are more impaired [14]. The prevalence of smoking, alcohol abuse and substance abuse is generally higher in TRS than non-TRS, and much higher than in the general population [15]. Across studies, 79% of patients with TRS show aggressive behaviors, and more than half have suicidal ideation or have attempted suicide [15]. From a caregiving perspective, there is a substantial burden on the informal carers of patients with schizophrenia and other psychotic disorders in terms of time, money, and psychological distress [16–18]. In focus groups, carers of patients with TRS – the majority of whom were family members – reported an average of 37 h per week providing direct care, as well as being on call much of the rest of the week, thereby affecting their own finances, career prospects, social relationships, and sense of freedom [18].

Neuroimaging studies investigating the underlying neurobiological reasons for resistance suggest that TRS may be a subtype of schizophrenia that is distinct from non-TRS, as shown by a greater reduction of grey matter, predominantly in frontal areas [19, 20]. TRS itself shows clinical heterogeneity in terms of course and outcome, whereby some patients respond to antipsychotics initially but develop resistance over time, and other patients never respond to antipsychotics [21]. This heterogeneity may reflect differences in underlying neurobiology, with hypotheses for TRS including normodopaminergic (as opposed to hyperdopaminergic) function [22], changes in glutamate neurotransmission [23], and the development of dopamine supersensitivity with the long-term administration of antipsychotics [24].

Using data from a survey of psychiatrists in the US, the aim of this study was to clinically characterize a TRS population, compared with a non-TRS population, in terms of patient demographic characteristics, burden of symptoms, treatment history, and factors influencing therapeutic choice. In addition, this study aimed to explore psychiatrists’ perceptions of medications used in TRS.

## Methods

This study comprised a 45-min online survey with psychiatrists in the US, conducted from 12th February 2017 to 16th March 2017. The target for recruitment was 200 respondents. Psychiatrists were eligible if they had been qualified for ≥ 3 years, were actively treating patients with TRS, were seeing ≥ 50 patients with schizophrenia per month, had ≥ 5 patients with TRS in their current caseload, prescribed atypical antipsychotics, were not on the payroll of a pharmaceutical company (excluding work as a speaker or involvement with clinical trials), and had not participated in any schizophrenia market research in the last month. A mix of hospital-/office-based and public/private psychiatrists were approached.

The survey is available as an online supplement (see Additional file 1). It comprised 1) an introduction; 2) a screening section that assessed psychiatrists for eligibility and asked them to spontaneously define TRS; 3) a section in which psychiatrists self-selected and completed patient records for three of their patients: two with TRS (definition below) and one with non-TRS (“[a] patient with schizophrenia who as per your clinical judgment is not classified as treatment resistant”); and 4) a section in which psychiatrists described their perceptions of antipsychotics and adjunctive psychotropic medications used in TRS. Patient information was anonymous, and no information was requested that would allow individuals to be identified. The survey was conducted in accordance with Market Research Society (MRS) and Council of American Survey Research Organizations (CASRO) guidelines [25, 26].

Section 3 of the survey (patient records) collected demographic and clinical characteristics for each patient, together with treatment history based on the last three antipsychotic treatment regimens (current therapy, one regimen previous, and two regimens previous), where a new regimen was defined as the switch between, or addition of, antipsychotic medication(s). Psychiatrists completed the patient records section based on a review of their own chart notes, and must have managed their selected patients within the past 6 months. Half of the psychiatrists were asked to select patient records based on their own ‘spontaneous’ definition of TRS, whereas the other half were asked to select patient records based on the following ‘prompted’ definition of TRS: “Schizophrenia patients for whom there is a lack of satisfactory improvement in clinical symptoms and/or functioning, despite sufficient duration of, and adherence to, therapeutic doses of at least two antipsychotic agents, one of which is an atypical (second-generation) agent”. This definition was adapted from treatment guidelines [2, 6–8]. The potential to pool the two TRS subpopulations (spontaneous and prompted definitions) into a single TRS group was investigated using classification and regression tree analysis (CART), discriminant analysis, and error count estimates across questions.

Statistical analyses were performed using Quantum v5.8 (IBM). *P*-values were calculated using the column means test. For patients’ current symptomatology, Pearson’s correlation coefficients were calculated for positive symptom severity versus negative symptom severity. All tests were two-sided, and alpha was set at 0.05 without correction for multiple testing.

## Results

### Psychiatrist demographics

Altogether, 2800 survey invitations were sent out. Of the invitees, 2186 (78.1%) did not respond, 410 (14.6%) responded but were deemed ineligible based on their answers to the screening questions, and 204 (7.3%) responded, were deemed eligible, and completed the survey.

Among the 204 psychiatrists who completed the survey, the mean (± standard deviation) time in practice as a qualified psychiatrist was 16.3 ± 7.3 years. The mean proportion of professional time spent in clinical practice (as opposed to in an academic or research setting) was 96.5 ± 5.1%. In the last 6 months, the mean schizophrenia caseload was 229.0 ± 179.0 patients, of whom 67.6 ± 76.8 (29.5%) had TRS. Outpatients comprised 82.1% of the schizophrenia caseload, and inpatients comprised 17.9%. Most psychiatrists (52.5%) saw the majority of their patients in an office/clinic; other locations were a community mental health center (34.8%), community hospital (6.4%), academic hospital (4.9%), or other setting (1.5%).

Compared to the eligible psychiatrists, ineligible psychiatrists (data for *n* = 399) had been in practice for a similar time (15.5 ± 10.6 years), spent less time in the clinic (92.4 ± 15.1%, *p* < 0.001), and had a smaller mean schizophrenia caseload (125.0 ± 147.5 patients, *p* < 0.001), of whom fewer patients had TRS (23.6 ± 37.1 [18.9%], *p* < 0.001). Ineligible psychiatrists had a higher proportion of inpatients in their caseload (33.7%, *p* < 0.001 versus eligible psychiatrists), and patients were seen in the following locations: office/clinic, 44.9%; community mental health center, 18.3% (*p* < 0.001); community hospital, 20.1% (*p* < 0.001); academic hospital, 12.3% (*p* = 0.004); or other setting, 4.5%.

### Psychiatrists’ spontaneous definitions of TRS

**Fig. 1:**
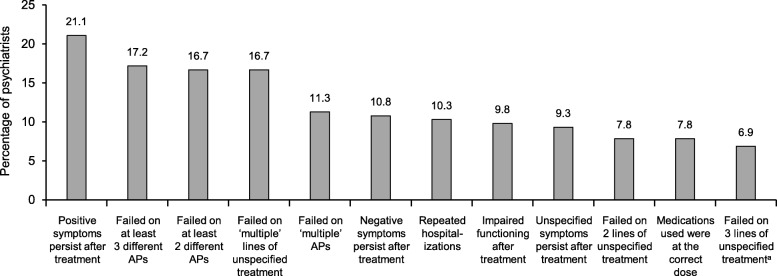
Spontaneous definitions of TRS: criteria stated by ≥ 5% of surveyed psychiatrists (*n* = 204). American Psychiatric Association definition of TRS: “Treatment resistance is defined as little or no symptomatic response to multiple (at least two) antipsychotic trials of an adequate duration (at least 6 weeks) and dose (therapeutic range)” [2]. ^a^Including at least two classes of treatment. AP = antipsychotic; TRS = treatment-resistant schizophrenia

All eligible psychiatrists were asked to enter a spontaneous definition of TRS into an open-ended text box. The criteria stated by ≥ 5% of psychiatrists are presented in Fig. 1. Although the majority of psychiatrists stated that patients must have failed prior treatments to be classified as treatment resistant, there was variation with regard to the number of treatment failures and the types of treatment on which they had failed (Fig. 1). Only 16.7% of psychiatrists specifically stated that patients must have failed on at least two different antipsychotics; equally common was failure on at least three different antipsychotics (17.2%), or failure on ‘multiple’ lines of unspecified treatment (16.7%). Just 7.8% of psychiatrists specified that medications must have been correctly dosed, and < 5% specified that medications must have been used for 6 weeks or an ‘adequate’ period of time, or that patients must have been adherent to the treatment. Persistent symptoms were a common component of the definitions, whether positive symptoms (21.1%), negative symptoms (10.8%), or unspecified symptoms (9.3%).

### TRS versus non-TRS patient reports: demographic and clinical characteristics

Using three different statistical approaches (CART, discriminant analysis, and error count estimates), it was not possible to reliably differentiate between the spontaneously defined TRS group and the group of patients defined following the prompted definition of TRS (data not shown). Due to their similarity and for simplicity in the analyses, these two groups were combined into a single TRS group.

Patient demographic and clinical characteristics for TRS versus non-TRS patients are presented in Table 1. TRS patients were, on average, around 3 years older than non-TRS patients (*p* = 0.001) and were more likely to be male (*p* = 0.034). TRS versus non-TRS patients were more likely to be unemployed (*p* < 0.001), less likely to live with a partner or family (*p* = 0.004), and more likely to live in a sheltered home (*p* < 0.001). Patients with TRS were more likely to have been hospitalized at least once than patients with non-TRS (93.4% versus 74.0%, *p* < 0.001) and were more likely to have attempted suicide (*p* < 0.001).

**Table 1:** Patient demographic and clinical characteristics

	TRS (*n* = 408)	Non-TRS (*n* = 204)	*p*-value
Age (years), mean (SD)	39.3 (11.3)	36.0 (11.8)	**0.001**
Distribution, % (*n*)			
< 18	0.0 (0)	0.5 (1)	0.16
18–24	6.4 (26)	15.2 (31)	**< 0.001**
25–34	32.6 (133)	37.7 (77)	0.21
35–44	25.2 (103)	25.0 (51)	0.95
45–54	22.8 (93)	10.3 (21)	**< 0.001**
55–64	12.0 (49)	10.3 (21)	0.53
≥ 65	1.0 (4)	1.0 (2)	0.50
Male, % (*n*)	73.0 (298)	64.7 (132)	**0.034**
Occupational status, % (*n*)			
Unemployed	74.5 (304)	45.1 (92)	**< 0.001**
Employed part-time	8.8 (36)	23.5 (48)	**< 0.001**
Employed full-time	5.4 (22)	11.8 (24)	**0.005**
In education/studying	4.9 (20)	9.8 (20)	**0.021**
Volunteering	4.4 (18)	7.8 (16)	0.081
Unknown	2.0 (8)	2.0 (4)	0.50
Marital status, % (*n*)			
Single	71.8 (293)	68.1 (139)	0.35
Divorced	13.5 (55)	12.3 (25)	0.67
Married/long-term relationship	10.0 (41)	15.2 (31)	0.063
Widowed	1.2 (5)	1.5 (3)	0.80
Unknown	3.4 (14)	2.9 (6)	0.75
Living arrangements, % (*n*)			
With a partner/family	40.9 (167)	53.4 (109)	**0.004**
In a sheltered home	29.4 (120)	10.8 (22)	**< 0.001**
Alone	20.1 (82)	26.0 (53)	0.099
With friends	5.4 (22)	5.9 (12)	0.80
No fixed address	2.9 (12)	1.5 (3)	0.27
Unknown	1.2 (5)	2.5 (5)	0.26
Age at symptom onset (years), mean (SD)	22.4 (6.0)	22.8 (5.9)	0.49
Age at diagnosis (years), mean (SD)	24.1 (5.8)	24.7 (6.5)	0.31
Time between symptom onset and diagnosis (years), mean (SD)	1.8 (2.3)	1.8 (3.0)	0.76
Hospitalization status, % (*n*)			
At least twice (but not currently)	72.5 (296)	39.7 (81)	**< 0.001**
Once only (but not currently)	13.2 (54)	31.4 (64)	**< 0.001**
Currently hospitalized (also in past)	6.4 (26)	2.5 (5)	**0.038**
Currently hospitalized (first time)	1.2 (5)	0.5 (1)	0.39
Never	4.9 (20)	23.5 (48)	**< 0.001**
Unknown	1.7 (7)	2.5 (5)	0.54
Number of hospitalizations (for patients with > 1 hospitalization), mean (SD)	(*n* = 322)	(*n* = 86)	
	7.2 (7.0)	5.7 (4.2)	0.090
Number of suicide attempts, mean (SD)	0.9 (1.5)	0.5 (1.3)	**0.004**
Distribution, % (*n*)			
0	50.0 (204)	69.1 (141)	**< 0.001**
1	15.4 (63)	9.3 (19)	**0.036**
2	14.0 (57)	3.4 (7)	**< 0.001**
3 or more	8.1 (33)	6.4 (13)	–
Unknown	12.5 (51)	11.8 (24)	0.79

*SD* standard deviation; *TRS* treatment-resistant schizophrenia

*p*-values in bold are < 0.05

**Fig. 2:**
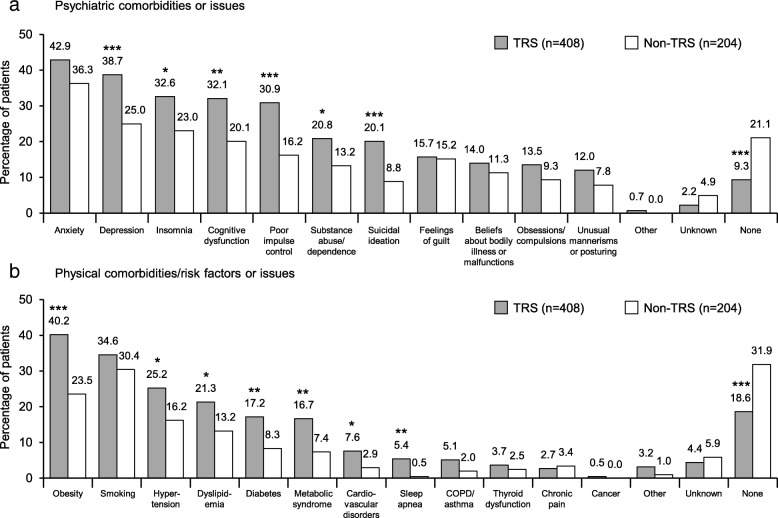
**a** Psychiatric and **b** physical comorbidities/risk factors or issues experienced at any point since schizophrenia diagnosis. *p < 0.05, **p < 0.01, ***p ≤ 0.001 versus non-TRS. COPD = chronic obstructive pulmonary disease; TRS = treatment-resistant schizophrenia

Various physical and psychiatric comorbidities/risk factors or issues were more common in TRS than non-TRS, including obesity, depression, insomnia, cognitive dysfunction, poor impulse control, and hypertension (all *p* < 0.05 and with incidence > 25% in TRS) (Fig. 2). The mean number of physical comorbidities per patient with TRS was 1.8 ± 1.7, and with non-TRS was 1.1 ± 1.2 (*p* < 0.05). The mean number of psychiatric comorbidities per patient with TRS and non-TRS was 2.7 ± 2.0 and 1.9 ± 1.8, respectively (*p* < 0.05).

Considering a patient’s current symptomatology, positive, negative, and cognitive symptoms were each more prevalent, more severe, and occurred more frequently, in TRS than non-TRS (Table 2). The most prevalent positive symptoms, in TRS and in non-TRS, were delusions (50.7% versus 35.3%, *p* < 0.001) and hallucinations (46.1% versus 24.5%, *p* < 0.001). Selecting from the patient’s current positive symptoms, psychiatrists considered delusions and hallucinations to be the most important to eliminate in order to improve long-term prognosis, both for patients with TRS and non-TRS. Correlation analysis of positive symptom severity versus negative symptom severity in patients with TRS showed a positive correlation of 0.54 between the two variables; in non-TRS the correlation was similar (0.58).

**Table 2:** Prevalence, severity and frequency of current schizophrenia symptoms

	TRS (*n* = 408)	Non-TRS (*n* = 204)	*p*-value
Positive symptoms			
Experiencing any, % (*n*)	84.6 (345)	64.2 (131)	**< 0.001**
Delusions	50.7 (207)	35.3 (72)	**< 0.001**
Hallucinatory behavior	46.1 (188)	24.5 (50)	**< 0.001**
Conceptual disorganization	24.3 (99)	17.2 (35)	**0.046**
Excitement or agitation	11.8 (48)	9.8 (20)	0.47
Hostility or aggression	11.5 (47)	4.9 (10)	**0.008**
Severity, mean (SD)^a^	3.0 (1.0)	2.5 (1.0)	**< 0.001**
Experienced on a daily basis, % (*n*)	27.5 (95)	9.2 (12)	**< 0.001**
Negative symptoms			
Experiencing any, % (*n*)	73.3 (299)	61.8 (126)	**0.004**
Blunted affect	37.0 (151)	30.9 (63)	0.14
Emotional withdrawal	35.8 (146)	26.5 (54)	**0.021**
Social withdrawal	30.1 (123)	21.6 (44)	**0.025**
Poor rapport	16.2 (66)	14.2 (29)	0.53
Severity, mean (SD)^a^	3.0 (1.0)	2.4 (0.9)	**< 0.001**
Experienced on a daily basis, % (*n*)	45.5 (136)	20.6 (26)	**< 0.001**
Cognitive symptoms			
Experiencing cognitive dysfunction, % (*n*)	37.3 (152)	24.0 (49)	**0.001**
Severity, mean (SD)^a^	3.0 (1.0)	2.5 (0.9)	**0.002**
Experienced on a daily basis, % (*n*)	61.2 (93)	36.7 (18)	**0.003**

^a^Severity rated 1 (minimal), 2 (mild), 3 (moderate), 4 (moderate–severe), 5 (severe), or 6 (extreme), for those patients experiencing symptoms

*SD* standard deviation; *TRS* treatment-resistant schizophrenia

*p*-values in bold are < 0.05

For patients who were currently symptomatic, the impact of their symptoms on social and functioning domains was rated as one of ‘absent’, ‘mild’, ‘moderate’, ‘marked’, ‘severe’, or ‘very severe’. In TRS compared with non-TRS, symptoms were more likely to have a marked to very severe impact on the following domains: 1) socially useful activities (including work and study): 38.4% (147/383) versus 15.9% (27/170), *p* < 0.001; 2) personal and social relationships: 35.2% (135/383) versus 14.7% (25/170), *p* < 0.001; 3) self-care: 17.5% (67/383) versus 8.8% (15/170), *p* = 0.008; 4) disturbing/aggressive behavior: 14.1% (54/383) versus 4.7% (8/170), *p* = 0.001.

Psychiatrists were less satisfied with the progress of TRS versus non-TRS patients in terms of overall improvement in symptomatology, behavior, and quality of life since diagnosis. Measured from 1 (not at all satisfied) to 10 (extremely satisfied), the mean satisfaction score was 5.9 ± 1.9 in TRS and 7.2 ± 1.8 in non-TRS (*p* < 0.001).

### TRS versus non-TRS patient reports: treatment patterns

**Fig. 3:**
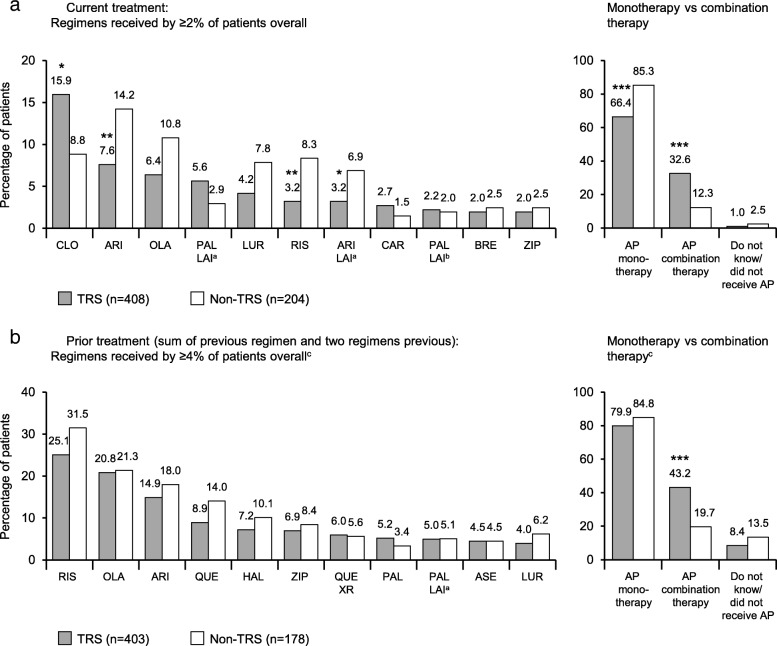
**a** Current and **b** prior treatment regimens in schizophrenia. ^a^1-monthly LAI. ^b^3-monthly LAI. ^c^Excluding patients who did not receive an antipsychotic at this line. *p < 0.05, **p ≤ 0.01, ***p < 0.001 versus non-TRS. AP = antipsychotic; ARI = aripiprazole; ASE = asenapine; BRE = brexpiprazole; CAR = cariprazine; CLO = clozapine; HAL = haloperidol; LAI = long-acting injectable; LUR = lurasidone; OLA = olanzapine; PAL = paliperidone; QUE = quetiapine; RIS = risperidone; TRS = treatment-resistant schizophrenia; XR = extended release; ZIP = ziprasidone

Considering all patients’ last three antipsychotic treatment regimens, the most common were oral monotherapy with risperidone (31.0% [187/604]), olanzapine (28.1% [170/604]), and aripiprazole (25.2% [152/604]). In TRS, clozapine monotherapy was the most common current regimen (15.9% [65/408]), though it was infrequently used in the prior two regimens (4.0% [16/403], summed across both regimens) (Fig. 3). Disease history was compared between the subgroup of TRS patients currently receiving clozapine (as monotherapy or in combination) and the subgroup not currently receiving clozapine. The mean age at symptom onset for those currently receiving clozapine was 21.9 ± 4.5 years, compared with 22.6 ± 6.4 years for those not receiving clozapine. The mean duration of disease (since diagnosis) for those currently receiving clozapine was 15.0 ± 10.4 years, compared with 13.7 ± 9.8 years for those not receiving clozapine.

Antipsychotic combination therapy (i.e., more than one concurrent antipsychotic medication) was more common in TRS than in non-TRS, in the current treatment regimen and in the prior two regimens (both *p* < 0.001) (Fig. 3). Furthermore, patients with TRS versus non-TRS were more likely to have been prescribed the following adjunctive psychotropic medications while in the care of the psychiatrist: a mood stabilizer, 43.6% (178/408) versus 23.0% (47/204), *p* < 0.001; an antidepressant, 38.0% (155/408) versus 26.5% (54/204), *p* = 0.005; and an anxiolytic, 30.1% (123/408) versus 19.1% (39/204), *p* = 0.004.

**Fig. 4:**
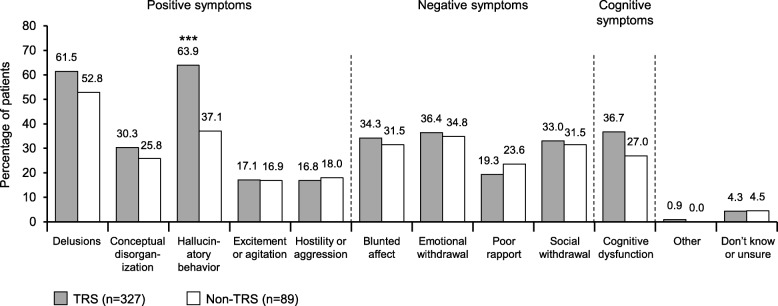
Schizophrenia symptoms leading to a treatment switch. ***p < 0.001 versus non-TRS. TRS = treatment-resistant schizophrenia

Antipsychotic switches were most commonly attributed to lack of efficacy (TRS, 71.4% [544/762]; non-TRS, 54.3% [164/302]; *p* < 0.001) and intolerability (TRS, 34.4% [262/762]; non-TRS, 38.4% [116/302]; *p* = 0.22) with the prior therapy. Of patients for whom inadequate control of positive, negative, or cognitive symptoms played a role in switching, persistent hallucinatory behavior was a driver for switching in 63.9% of TRS patients versus 37.1% of non-TRS patients (*p* < 0.001) (Fig. 4).

Over the prior two treatment regimens, there was no difference between TRS (*n* = 395) and non-TRS (*n* = 167) in the percentage of time that the patient was perceived to be adherent to their antipsychotic regimen (based primarily on patient and carer/family/nurse reports): typical antipsychotics (oral), 69.8% versus 63.3%, *p* = 0.32; atypical antipsychotics (oral), 69.7% versus 71.3%, *p* = 0.52; long-acting injectable (LAI) antipsychotics, 72.2% versus 78.2%, *p* = 0.22.

### Psychiatrists’ perceptions of medications used in TRS

The psychiatrists were asked to rank the order in which they would use ten specific treatment options to manage a patient with schizophrenia who had failed on two prior antipsychotics. The options were ranked as follows (mean rankings shown in parentheses): increase the dose of current antipsychotic (if tolerated) (1.6); add a second antipsychotic (3.6); suggest an LAI formulation (4.1); add a mood stabilizer (4.6); suggest clozapine (4.9); suggest switching to a (new) atypical antipsychotic (excluding clozapine) (5.0); add an antidepressant (5.6); add a third antipsychotic (5.9); suggest switching to a (new) typical antipsychotic (6.3); add an anxiolytic medication (6.3).

Considering all the patients with TRS in their care, on average, the psychiatrists said that 49.5% of their patients received antipsychotic monotherapy and 49.7% received antipsychotic combination therapy, with 0.8% receiving some other, unspecified approach. For monotherapy, 25.2% received a typical antipsychotic and 73.9% an atypical antipsychotic (other: 1.0%). For combination therapy, 12.2% were treated with two or more typical antipsychotic medications, 45.7% were treated with two or more atypical antipsychotic medications, and 40.9% were treated with a combination of one or more typical antipsychotics plus one or more atypical antipsychotics (other: 1.3%).

When the psychiatrists were asked to select their top antipsychotics from a comprehensive list in terms of their overall satisfaction with them in the context of treating/managing TRS (as monotherapy or combination therapy), the following antipsychotics were most commonly selected in the top three: clozapine (49.0%, 100/204); aripiprazole (43.1%, 88/204); olanzapine (41.7%, 85/204); paliperidone palmitate 1-monthly LAI (39.2%, 80/204); and aripiprazole 1-monthly LAI (30.9%, 63/204).

When asked to define ‘satisfactory improvement’ in the general management of schizophrenia, in addition to reduced symptomatology, improved self-care (41.2%, 84/204) and positive impact on social activities (25.0%, 51/204) were the most frequently stated markers of satisfactory improvement.

## Discussion

This online survey revealed that there is a lack of clarity among US psychiatrists regarding the definition of TRS, based on a relatively small sample of selected psychiatrists. For example, the psychiatrists’ spontaneous definitions varied with regard to the number of treatment lines (two, three, or multiple) and the types of treatment (antipsychotics or unspecified) on which a patient must have failed to be classified as having TRS. A lack of clarity regarding specific criteria was also noted in a recent consensus statement on TRS, which showed that 50% of TRS studies did not use operationalized criteria for TRS, and only 5% of studies used identical criteria [8]. Hence, further education on TRS may be needed for clinicians and researchers alike, in order to improve communication, as well as to aid the appropriate and timely identification of this patient group associated with a high need for additional treatments and improved outcomes.

Despite differences between the prompted definition of TRS and the participating psychiatrists’ spontaneous definitions, patients in these subgroups had, on average, similar demographic and clinical characteristics, which allowed pooling of the data. The similarity of these two subgroups suggests that it may not always be necessary to require a patient to meet all TRS criteria for them to be perceived as treatment resistant by psychiatrists. Alternatively, psychiatrists may have selected patients who were more resistant than the minimum requirements of their definitions. TRS is likely to be a heterogeneous condition, as shown in studies of clozapine: despite being the only currently approved medication for TRS, clozapine is associated with response in just 40.1% (95% confidence interval: 36.8 to 43.4%) of patients with TRS [27].

When psychiatrist-selected TRS and non-TRS patient records were compared, observations were in line with previous data suggesting that symptoms have a higher burden on patients with TRS than non-TRS, being associated with more unemployment, hospitalization, suicidality, and comorbidities [14, 15]. Positive, negative, and cognitive symptoms occurred with greater severity and frequency in TRS patients, and had a greater impact on various social and functioning domains, as seen in a previous study [14]. Of note, in the present study, persistent schizophrenia symptoms had a greater impact on self-care and disturbing/aggressive behavior in TRS than in non-TRS, underscoring the importance of identifying and addressing TRS early and effectively in order to improve overall outcomes.

Considering treatment patterns in the psychiatrist-selected patient records, the most widely used antipsychotics in the total population (TRS plus non-TRS) were risperidone, olanzapine, and aripiprazole. This result is broadly reflective of treatment patterns observed in the US [28], strengthening the results by showing external validity, despite the selected sample of survey responders.

In TRS, clozapine monotherapy was the most common current antipsychotic treatment, received by 15.9% of patients. This was higher than expected (based on US Medicaid claims data from 2001 to 2005, clozapine was initiated in only 5.5% of episodes for patients whose service-use patterns indicated TRS [29]), and may reflect selection bias 1) towards the inclusion of psychiatrists who focus on TRS and tend to use clozapine; and 2) towards psychiatrists selecting their clozapine patients for the study as they are easily identified and correctly treated for TRS as per guidelines. However, the majority of TRS patients did not receive clozapine, despite clozapine being the only pharmacological treatment approved for use in TRS and the recommended first-line TRS treatment in clinical guidelines from the American Psychiatric Association (APA), National Institute for Health and Care Excellence (NICE), and World Federation of Societies of Biological Psychiatry (WFSBP) [2, 6, 7].

Clozapine was the antipsychotic that the psychiatrists most commonly ranked in the top three for treating/managing TRS, though it was only ranked in the top three by 49.0% of psychiatrists. Moreover, out of ten different strategies to manage TRS, clozapine use was ranked fifth, with a preference to increase the dose of current antipsychotic, add a second antipsychotic, suggest an LAI formulation, and add a mood stabilizer, before initiating clozapine. This finding of a relatively low preference level of psychiatrists to use clozapine, the only evidence-based treatment strategy for TRS, in patients who they themselves consider to have TRS reflects previous research showing that the introduction of clozapine in TRS is often delayed by several years [30, 31], and that clozapine is underutilized in many countries [32]. Reasons for clozapine underutilization include concerns about its safety and tolerability, and the requirement for long-term blood testing [33, 34].

Despite being the top-ranked strategy to manage TRS in this study, there is limited evidence that antipsychotic dose escalation yields satisfactory results in schizophrenia patients with insufficient response to antipsychotic treatment [35, 36].

Antipsychotic combination therapy, ranked second by the psychiatrists, was more common in the TRS than non-TRS patient records (32.6% versus 12.3% in the current regimen). Considering all the patients with TRS in their care, the psychiatrists reported that as many as 49.7% received antipsychotic combination therapy. The use of antipsychotic combination therapy in schizophrenia, particularly in more severely ill and less treatment-responsive patients, is well documented [37, 38], and antipsychotic combination therapy is often used in preference to clozapine [33]. However, a recent meta-analysis of antipsychotic augmentation studies indicated that – in high-quality, double-blind studies in schizophrenia – antipsychotic combination therapy was not more efficacious than monotherapy for total psychopathology or positive symptoms, and that benefits were seen only on negative symptoms for the combination of a partial D_2_ agonist with a full D_2_ antagonist [39].

Similarly, in the present study, patients with TRS were more likely to have been prescribed adjunctive psychotropic medications, reflecting their more severe symptomatology. While it is common practice to combine antipsychotics with other psychotropic medications in cases of partial response or TRS, evidence for the efficacy of such strategies is inconsistent, despite multiple meta-analyses examining these combinations [40].

Ranked as the third best strategy to manage TRS by the psychiatrists, LAI treatment has recently been suggested as an optimal step before TRS can reliably be concluded [8]. There are no available data on the response rates of patients with TRS who start on LAIs versus staying on their prior regimen; however, recent studies in patients provisionally diagnosed with TRS showed that 35–44% had undetectable or subtherapeutic antipsychotic plasma levels, suggesting that these patients may be under-treated rather than treatment resistant [41, 42].

In the present study, psychiatrists estimated that the TRS and non-TRS populations were likely to be adherent to their antipsychotic regimen for approximately 70% of the time, based primarily on patient and informant reports. Prior data indicate that psychiatrists overestimate adherence in patients with schizophrenia [43]. Other researchers have reported reduced adherence in TRS versus non-TRS [14]; however, the psychiatrists surveyed in the present study did not perceive any difference in adherence rates between TRS and non-TRS samples, possibly due to overestimation of adherence in TRS patients.

Uncontrolled hallucinatory behavior was a clear driver for treatment change in the TRS group, differentiating TRS patients from non-TRS patients, and thereby highlighting an unmet treatment need in this population. Although negative and cognitive symptoms were prevalent and have been closely linked to functional disability [44], the result that hallucinations were the most common reason for an antipsychotic switch in TRS may be because current antipsychotics are more effective for treating positive symptoms than other symptom domains of schizophrenia [45–47].

Results of the present study need to be interpreted within its limitations. Questionnaires are, by their nature, limited with regard to validity, respondents’ comprehension of questions, and the reliability of respondents’ answers, including their judgment of the prevalence and severity of negative and cognitive symptoms. Recruitment did not specifically target psychiatrists working in the public sector, where most patients with schizophrenia (and, by extension, TRS) are seen [48]. Furthermore, the response rate to the questionnaire was low, the participating psychiatrists may have been a selected subgroup, and their choice of patients to include may not be representative of their caseload. With regard to psychiatrist selection, those who were eligible had a much higher mean schizophrenia caseload (229.0 versus 125.0 patients) and TRS caseload (67.6 versus 23.6 patients) compared with those who were ineligible, probably driven by the psychiatrist inclusion criteria. The reported treatment histories were limited because the survey considered only a patient’s last three treatment trials, though it is likely that many of them had a longer treatment history. Nonetheless, this study clinically characterized TRS in a large sample (over 400 TRS patient records), and the use of a questionnaire allowed the exploration of psychiatrists’ general perceptions, as well as providing patient-level data.

## Conclusions

According to the treating psychiatrists who took part in this study, symptoms have a greater clinical burden on patients with TRS than non-TRS. TRS was commonly managed by increasing the antipsychotic dose, antipsychotic combination therapy, starting an LAI, or adding a mood stabilizer, with initiating clozapine and switching antipsychotics ranked fifth and sixth, despite clozapine being the only approved treatment for TRS. Persistent hallucinations and delusions were the main clinical drivers for treatment change in TRS, with persistent hallucinations being considerably more common as a driver for treatment change in TRS than non-TRS. These data suggest that there is a need for new effective and tolerable treatments for patients who do not respond to available antipsychotics.

## Supplementary information

**Additional file 1.** A copy of the survey.

## Data Availability

The datasets generated and/or analyzed during the current study are not publicly available because no suitable repository for these data exists, but are available from the corresponding author on reasonable request.

## Abbreviations

APA: American Psychiatric Association
CART: Classification and regression tree analysis
CASRO: Council of American Survey Research Organizations
LAI: Long-acting injectable
MRS: Market Research Society
NICE: National Institute for Health and Care Excellence
TRS: Treatment-resistant schizophrenia
WFSBP: World Federation of Societies of Biological Psychiatry
