# Supplementary material for: Patient characteristics, burden and pharmacotherapy of treatment-resistant schizophrenia: results from a survey of 204 US psychiatrists
Source: BMC Psychiatry. 2019 Nov 14;19:362. doi: 10.1186/s12888-019-2318-x (PMC6857350; doi:10.1186/s12888-019-2318-x)
Supplement: Supplementary file 1 — Additional file 1. A copy of the survey. [file 12888_2019_2318_MOESM1_ESM.pdf]

Appendix. Copy of the survey

Appendix to:

Characteristics, burden and pharmacotherapy of treatment-resistant schizophrenia: results from a survey of 204 US psychiatrists.

Christoph U. Correll, Thomas Brevig, Cecilia Brain.

Introduction

Thank you for your interest in taking part in this survey.

We are currently conducting market research with a range of healthcare professionals on behalf of a pharmaceutical company to understand your current practice and perceptions with regard to schizophrenia.

The survey will last approximately 45 minutes.

Adherence to market research code of conduct:

This online questionnaire will be conducted in accordance with:

- Market Research Society (MRS) guidelines
- Council of American Survey Research Organizations (CASRO) guidelines

The aim of this research is to gain your views for market research purposes only and is not intended to be promotional.

Anything you see or read during this research should be treated as confidential.

The identity of respondents is confidential and none of your details will be passed on to any third party.

Any information you disclose will be treated in the strictest confidence and the results of the research will be aggregated to provide an overall picture of attitudes to the areas being covered in this survey. No answers will be attributable to you as an individual. The aggregated findings will be used for market research purposes. The aggregated findings may also be shared with third parties, working on behalf of the sponsoring pharmaceutical company.

The outputs of this research may be used by the sponsoring company in a promotional or external context at an aggregated level or using anonymized quotes.

You have the right to withdraw from the research at any time and to withhold information as you see fit.

Screening

We will now ask a few questions to see whether you are eligible to participate in the study, and if so you will move on to the main questionnaire.

SQ1

What is your primary medical specialty?

[Select one]

- Psychiatrist (adult patients)
- Child psychiatrist
- Primary care
- Neurologist
- Other

SQ2

For how long have you been qualified and practicing as a psychiatrist?

[Type answer]

__ years

SQ3

Within the last 5 years have you been on the payroll of a pharma company (excluding work as a speaker or involvement with clinical trials)?

[Select one]

- Yes
- No

SQ4

Are you board certified or board eligible in your specialty?

[Select one]

- Yes
- No

SQ5

Approximately what proportion of your professional time is spent in clinical practice (as opposed to in an academic or research setting)?

[Type answer]

__ %

SQ6

[Does not apply to the US]

SQ7

How many patients with schizophrenia have you managed within the last 6 months?

[Type answer]

__ patients with schizophrenia managed in the last 6 months

SQ7a

How many patients with schizophrenia do you see in an average month? Please specify the number of new and established patients seen.

[Type answer]

- New patients
- Established patients

SQ7b

Of the [_] patients you manage in an average month, what proportion are inpatients and what proportion are outpatients?

[Type answer]

- Inpatients
- Outpatients

SQ8

Where do you see the majority of your patients diagnosed with schizophrenia?

[Select one]

- Office/clinic
- Community hospital
- Academic hospital
- Community mental health center
- Other

SQ9a

Thinking about the schizophrenia patients who you have managed within the last 6 months, how many have treatment-resistant schizophrenia (TRS)?

[Type answer]

__ patients within my caseload have treatment-resistant schizophrenia (TRS)

SQ9b

In your clinical practice, how do you define treatment-resistant schizophrenia (TRS)? Please enter as much detail as possible in the box below

[Type answer]

SQ10

Which of the following treatments have you prescribed in the past 3 months for your patients with schizophrenia?

[Select all that apply]

Atypical antipsychotics – Orals

- Aripiprazole (Abilify)
- Asenapine (Saphris)
- Clozapine (Clozaril/Leponex)
- Brexpiprazole (Rexulti)
- Cariprazine (Vraylar)
- Iloperidone (Fanapt)
- Lurasidone (Latuda)
- Olanzapine (Zyprexa)
- Paliperidone (Invega)
- Quetiapine (Seroquase/Seroquel/Xeroquel)
- Quetiapine XR (Seroquase/Seroquel XR)
- Risperidone (Risperdal)
- Ziprasidone (Geodon)

Atypical antipsychotics – LAIs

- Aripiprazole (Abilify Maintena)
- Aripiprazole lauroxil (Aristada)
- Olanzapine pamoate monohydrate (Zyprexa Relprevv)
- Risperidone (Risperdal Consta)
- Paliperidone palmitate (Invega Sustenna)
- Paliperidone palmitate (Invega Trinza)

Typical antipsychotics – Orals

- Chlorpromazine
- Haloperidol (Haldol)
- Levomepromazine
- Zuclopenthixol hydrochloride (Clopixol)

- Fluphenazine
- Flupentixol
- Other (please specify)

Typical antipsychotics – LAIs

- Haloperidol decanoate
- Zuclopenthixol decanoate
- Fluphenazine decanoate
- Flupentixol decanoate

Other

- None of the above

Section 1: Patient record forms

Thank you: we're happy to confirm that you are eligible to participate in this research.

For this first section, we will be asking for some information about your patients with schizophrenia. Please be assured that this information will remain anonymous, and we will not request any information that will allow these individuals to be identified.

For research purposes, we request that the information provided is accurate, and therefore ask you to refer to your own records, in order to provide as much detail as possible.

If you need time to access or collate this information, you can pause this survey and re-access it at any time.

We would like you to answer the following questions for three different patients:

- [For 50% of responders] Two patients with treatment-resistant schizophrenia (TRS), who you have managed within the last 6 months. If possible, please consider established patients whose treatment history you are familiar with, and will be able to answer questions on in detail.
- [For 50% of responders] Two patients with schizophrenia, who match the following definition:
  "Schizophrenia patients for whom there is a lack of satisfactory improvement in clinical symptoms and/or functioning, despite sufficient duration of, and adherence to, therapeutic doses of at least two antipsychotic agents, one of which is an atypical (second-generation) agent".
  Please select such patients who you have managed within the last 6 months. If possible, please consider established patients whose treatment history you are familiar with, and will be able to answer questions on in detail.
- [All responders] And one patient with schizophrenia (who as per your clinical judgment is not classified as treatment resistant). Please simply consider a patient that you have seen within the last month, who does not have treatment-resistant schizophrenia (TRS) based on your clinical opinion. Again, please consider an established patient whose treatment history you are familiar with, and will be able to answer questions on in detail.

Demographics

Q1

Patient's age

[Type answer]

__ years old

Q2

Patient's gender

[Select one]

- Male

- Female

Q3a

To the best of your knowledge, what is this patient's occupational status?

[Select one]

- Employed (full-time)
- Employed (part-time)
- In education/studying (full- or part-time)
- Working in a volunteer capacity
- Unemployed
- Don't know/unsure

Q3b

To the best of your knowledge, what is this patient's marital status?

[Select one]

- Single
- Married/in a long-term relationship
- Divorced
- Widowed
- Don't know/unsure

Q3c

Which of the following best describes this patient's home life?

[Select one]

- Patient lives alone (rented or owned accommodation)
- Patient lives with others (spouse/partner/family, rented or owned accommodation)
- Patient lives with others (friends/other, rented or owned accommodation)
- Patient lives in sheltered home/group home/supported living
- Patient has no fixed address/is homeless
- Don't know/unsure

Q3d

Which of the following statements best describe the frequency of contact that this patient has with the following groups/individuals, in terms of the provision of ongoing support (social, domestic, financial, etc.)?

[Select one per row]

	Never	A few times a year	Once every few months	Around once a month	3–4 times a month	2–3 times a week	On a daily basis	Don't know
Social or case worker/ ACT team								
Family members								
Partner or spouse								
Friends								
Non-profit charitable organizations								

Q3e

Which of the following statements best describes the role that the following groups play in the management of this patient's treatment for schizophrenia?

[Select one per Q3d row, for rows 1–4, as applicable]

- No role in decision making regarding their treatment for schizophrenia
- A limited role in decision making regarding their treatment for schizophrenia (i.e., occasionally attend appointments, provide some feedback on patient's symptoms/progress)
- An important role in decision making regarding their treatment for schizophrenia (i.e., attend most appointments, provide regular feedback on patient's symptoms/progress, can feed into decision making for treatment decisions)
- An essential role in decision making regarding their treatment for schizophrenia (i.e., attend all appointments, provide detailed feedback on patient's symptoms/progress, regularly help drive decision making for treatment decisions)
- Unsure/don't know

Q4

Which of the following best describes this patient's occupation?

[Select one, if employed]

- Works in a skilled/senior professional or commercial role (e.g., office-based profession, academic/medical role)
- Works in a mid-level/junior professional or commercial role (e.g., office-based profession)
- Works in a public service role (e.g., bus/taxi driver, emergency services, administrative worker, etc.)

- Works in a catering, tourism or entertainment role (e.g., restaurant worker, bartender, theater or cinema worker)
- Works in manual labor role (e.g., cleaner, plumber, electrician, builder)
- Student/in training
- Other
- Don’t know/unsure

Patient’s overall history and treatment

Q5a

To the best of your knowledge, at what age did the patient first experience the onset of schizophrenia symptoms?

[Type answer or select ‘Don’t know/unsure’]

__ years old

Q5b

To the best of your knowledge, at what age was this patient actually diagnosed with schizophrenia?

[Type answer or select ‘Don’t know/unsure’]

__ years old

Q5ci

[Ask if responder was allocated to textbook definition of TRS]

Do you feel that this patient has treatment-resistant schizophrenia (TRS), in line with your professional experience and understanding of this condition?

[Select one]

- Yes
- No

Q5cii

[Ask if TRS]

To the best of your knowledge, at what age was this patient classified as having treatment-resistant schizophrenia (TRS)?

[Type answer or select ‘Don’t know/unsure’]

__ years old

Q5d

[Ask if TRS]

Which of the following best describes the onset of this patient's treatment-resistant schizophrenia (TRS)?

[Select one]

- Patient was classified as having TRS from their first treatment trial (i.e., has never responded to therapy)
- Patient was classified as having TRS from their first relapse (i.e., responded to first treatment trial, then lost response)
- Patient responded to more than one treatment, but was classified as having TRS within the first 5 years of treatment
- Patient responded to more than one treatment, but was classified as having TRS after more than 5 years of treatment
- Don't know/unsure

Q6a

Which of the following statements best describes this patient's experience regarding hospitalization?

[Select one]

- Patient has never been hospitalized for schizophrenia
- Patient has been hospitalized for schizophrenia (one hospitalization only) but is not currently hospitalized
- Patient has been hospitalized for schizophrenia at least twice but is not currently hospitalized
- Patient is currently hospitalized (first hospitalization)
- Patient is currently hospitalized (has been hospitalized in the past)
- Don't know/unsure if patient has been hospitalized

Q6b

To the best of your knowledge, how many hospitalizations due to schizophrenia has this patient had, in total?

[Type answer, if applicable, or select 'Don't know/unsure']

__ hospitalizations

Q6c

To the best of your knowledge, how many times (if any) has the patient attempted to commit suicide?

[Type answer or select 'Don't know/unsure']

__ suicide attempts

Q7a

We would like to know which pharmacological treatments this patient has received at different points in time. By selecting the relevant product (or products) in each column, tell us their history chronologically, by recording this patient's:

1. Current therapy regimen
2. Therapy regimen received immediately prior to this current regimen (i.e., one regimen previously)
3. Therapy regimen received most recently before this prior regimen (i.e., two regimens previously)

For this exercise, please define a new "regimen" as the switch between, or addition of, antipsychotic medication.

If this patient is being treated (or has been treated) with multiple therapies at a given time (i.e., polypharmacy), please indicate this by selecting multiple treatment options in the relevant column.

For each regimen we will ask you to outline the different dosages of each medication that the patient received (if applicable).

If the patient did not receive a treatment at a particular line, please choose this option below.

[Select all that apply]

	1. Current therapy regimen	2. Therapy regimen received immediately prior to this current regimen	3. Therapy regimen received before this prior regimen
**Atypical antipsychotics – Orals**			
Aripiprazole (Abilify)			
Asenapine (Saphris)			
Clozapine (Clozaril/Leponex)			
Brexpiprazole (Rexulti)			
Cariprazine (Vraylar)			
Iloperidone (Fanapt)			
Lurasidone (Latuda)			
Olanzapine (Zyprexa)			
Paliperidone (Invega)			
Quetiapine (Seroquase/Seroquel/Xeroquel)			
Quetiapine XR (Seroquase/Seroquel XR)			
Risperidone (Risperdal)			
Ziprasidone (Geodon)			

**Atypical antipsychotics – LAIs**			
Aripiprazole (Abilify Maintena)			
Aripiprazole lauroxil (Aristada)			
Olanzapine pamoate monohydrate (Zyprexa Relprevv)			
Risperidone (Risperdal Consta)			
Paliperidone palmitate (Invega Sustenna)			
Paliperidone palmitate (Invega Trinza)			
**Typical antipsychotics – Orals**			
Chlorpromazine			
Haloperidol (Haldol)			
Levomepromazine			
Zuclopenthixol hydrochloride (Clopixol)			
Fluphenazine			
Flupentixol			
Other (please specify)			
**Typical antipsychotics – LAIs**			
Haloperidol decanoate			
Zuclopenthixol decanoate			
Fluphenazine decanoate			
Flupentixol decanoate			
**Other**			
Don't know/unsure of therapy received			
Did not receive therapy at this line			

Q7ai

Considering this patient's current therapy regimen: please enter the dosages (in mg) you prescribed for each treatment, indicating:

- The starting dose
- The maximum dose prescribed

- The current dose
- Time taken to titrate up to the maximum dose prescribed

Please note: 3+ weeks must be entered in months + weeks

[Type answer or select 'Don't know/unsure']

**Current therapy regimen**				
	**Starting dose**	**Maximum dose prescribed**	**Current dose**	**Time taken to titrate up to the maximum dose prescribed**
[Drug(s) selected in Q7a]	__ mg	__ mg	__ mg	__ years __ months __ weeks

Q7aii

Considering the therapy regimen this patient received immediately prior to their current regimen: please enter the dosages (in mg) you prescribed for each treatment, indicating:

- The starting dose
- The maximum dose prescribed
- The predominant dose
- Time taken to titrate up to the maximum dose prescribed

Please note: 3+ weeks must be entered in months + weeks

[Type answer or select 'Don't know/unsure']

**Therapy regimen received immediately prior to this current regimen (i.e., one regimen previously)**				
	**Starting dose**	**Maximum dose prescribed**	**Predominant dose**	**Time taken to titrate up to the maximum dose prescribed**
[Drug(s) selected in Q7a]	__ mg	__ mg	__ mg	__ years __ months __ weeks

Q7aiii

Considering the therapy regimen this patient received before this prior regimen: please enter the dosages (in mg) you prescribed for each treatment, indicating:

- The starting dose
- The maximum dose prescribed
- The predominant dose
- Time taken to titrate up to the maximum dose prescribed

Please note: 3+ weeks must be entered in months + weeks

[Type answer or select 'Don't know/unsure']

**Therapy regimen received most recently before this prior regimen (i.e., two regimens previously)**				
	**Starting dose**	**Maximum dose prescribed**	**Predominant dose**	**Time taken to titrate up to the maximum dose prescribed**

[Drug(s) selected in Q7a]	__ mg	__ mg	__ mg	__ years __ months __ weeks

Q7b

For how long was/has this patient [been] treated with each of these therapy regimens?

Current therapy regimen	__ years __ months__ weeks
Prior therapy regimen	__ years __ months__ weeks
Two regimens previously	__ years __ months__ weeks

[Type answer for each row, if applicable, or select 'Don't know/unsure']

Q8a

Considering each of these therapy regimens: during the time period that is clinically relevant for the outcome of the treatment, for what percentage of the time was/is this patient adherent to therapy?

Current therapy regimen	__ % of the time
Prior therapy regimen	__ % of the time
Two regimens previously	__ % of the time

[Type answer for each row, if applicable, or select 'Don't know/unsure']

Q8b

Considering each of these therapy regimens: how has adherence been measured/monitored for this patient? Please select all that apply.

	Current therapy regimen	Prior therapy regimen	Two regimens previously
Asking patient for a report on their adherence			
Asking carer/family/nurse for a report on the patient's adherence			
Reviewing prescription records or asking their pharmacist			
Pill count			
Measuring medication level (i.e., bloodwork)			
Other monitoring/measurement (please specify)			
None of the above			
Don't know/unsure			

Q8c

Which of the following, if any, have you attempted/used to try and address this patient's adherence? Please select all that apply.

	Current therapy regimen	Prior therapy regimen	Two regimens previously
Supervised medication intake			
Suggested a long-acting injectable therapy			
Use of a long-acting injectable therapy			
Cognitive behavioral therapy or other psychological intervention			
Other solution (please specify)			
None of the above			
Don't know/unsure			

Q9a

We would like to understand the reasons why this patient changed therapy regimens over time. For each treatment change, please indicate the main reason(s) for this change. Up to three reasons can be selected for each treatment change.

	Reason for change from [_] to [_] (prior treatment regimen)	Reason for change from [_] to [_] (current regimen)
Side effects experienced with prior therapy		
Prior therapy not having an adequate effect on symptom reduction		
No response to therapy		
Insurance or cost issues with prior therapy		
Request for other medication with alternative dosing		
Patient not taking prior therapy as specified in treatment plan		
Need for an alternative formulation (e.g., needed long-acting injectable option)		
Patient's evolving disease symptomology/disease profile		
Breakthrough symptomology (not non-adherence related)		

Patient's (or caregiver's) request		
Patient's negative attitude towards medication		
Caregiver's negative attitude towards medication		
Stigma of psychiatric medications		
Patient's lack of insight into having schizophrenia and/or treatment need		
Other reason for change (please specify)		
Don't know/unsure		

Q10a

[Ask if "Side effects experienced with prior therapy" was selected for Q9a (change to prior treatment regimen)]

You mentioned that side effects played a role in changing treatment from [_] to [_]. Which particular side effect(s) were involved? Please select all that apply.

- Extrapyramidal: acute dystonia
- Extrapyramidal: akathisia
- Extrapyramidal: parkinsonism
- Extrapyramidal: dyskinesia
- Extrapyramidal: withdrawal dyskinesia
- Sedation
- Prolactin/sexual side effects
- Metabolic side effects (please specify)
- Orthostatic hypertension
- Other (please specify)
- Don't know/unsure

Q10b

[Ask if "Prior therapy not having an adequate effect on symptom reduction" was selected for Q9a (change to prior treatment regimen)]

You mentioned that inadequate symptom control played a role in changing treatment from [_] to [_]. Which particular symptoms were not adequately controlled and led to the requirement for treatment change? Please select all that apply.

Positive symptoms

- Delusions
- Conceptual disorganization

- Hallucinatory behavior
- Excitement/agitation
- Hostility/aggression

Negative symptoms

- Blunted affect
- Emotional withdrawal
- Poor rapport
- Social withdrawal

Cognitive symptoms

- Cognitive dysfunction

Other

- Other symptoms (please specify)
- Don't know/unsure

Q10bi

[Ask if any positive symptom was selected in Q10b]

How would you rate this patient's positive symptomology at the time of treatment change?

[Select one]

- Minimal – questionable, subtle or suspected pathology
- Mild – symptom's presence clearly established but not pronounced
- Moderate – symptom represents serious problem but occurs occasionally or only intrudes on daily life to a moderate extent
- Moderate severe – manifestations that distinctly impact on functioning but are not 'all-consuming' and can be contained at will
- Severe – symptom presents frequently, is highly disruptive to patient's life, and calls for direct supervision
- Extreme – most serious level of psychopathology where manifestations drastically interfere with life functions and close supervision is required

Q10bii

[Ask if any negative symptom was selected in Q10b]

How would you rate this patient's negative symptomology at the time of treatment change?

[Select one]

- Minimal – questionable, subtle or suspected pathology
- Mild – symptom's presence clearly established but not pronounced

- Moderate – symptom represents serious problem but occurs occasionally or only intrudes on daily life to a moderate extent
- Moderate severe – manifestations that distinctly impact on functioning but are not ‘all-consuming’ and can be contained at will
- Severe – symptom presents frequently, is highly disruptive to patient’s life, and calls for direct supervision
- Extreme – most serious level of psychopathology where manifestations drastically interfere with life functions and close supervision is required

Q10biii

[Ask if cognitive dysfunction was selected in Q10b]

How would you rate this patient’s cognitive symptomology at the time of treatment change?

[Select one]

- Minimal – questionable, subtle or suspected pathology
- Mild – symptom’s presence clearly established but not pronounced
- Moderate – symptom represents serious problem but occurs occasionally or only intrudes on daily life to a moderate extent
- Moderate severe – manifestations that distinctly impact on functioning but are not ‘all-consuming’ and can be contained at will
- Severe – symptom presents frequently, is highly disruptive to patient’s life, and calls for direct supervision
- Extreme – most serious level of psychopathology where manifestations drastically interfere with life functions and close supervision is required

Q10c

[Ask if “Prior therapy not having an adequate effect on symptom reduction” was selected for Q9a (change to prior treatment regimen)]

How would you describe the level of severity of this patient’s symptoms in terms of impact on the following domains at the time of treatment change from [_] to [_]?

Q10ci Socially useful activities, including work and study

[Select one]

- Absent
- Mild
- Manifest
- Marked
- Severe
- Very severe
- Don’t know

Q10cii Personal and social relationships

[Select one]

- Absent
- Mild
- Manifest
- Marked
- Severe
- Very severe
- Don't know

Q10ciii Self-care

[Select one]

- Absent
- Mild
- Manifest
- Marked
- Severe
- Very severe
- Don't know

Q10civ Disturbing and aggressive behaviors

[Select one]

- Absent
- Mild
- Manifest
- Marked
- Severe
- Very severe
- Don't know

Q11a

[Ask if "Side effects experienced with prior therapy" was selected for Q9a (change to current treatment regimen)]

You mentioned that side effects played a role in changing treatment from [_] to [_] (current regimen). Which particular side effect(s) were involved? Please select all that apply.

- Extrapyramidal: acute dystonia
- Extrapyramidal: akathisia
- Extrapyramidal: parkinsonism
- Extrapyramidal: dyskinesia

- Extrapyramidal: withdrawal dyskinesia
- Sedation
- Prolactin/sexual side effects
- Weight gain
- Metabolic side effects (please specify)
- Orthostatic hypertension
- Other (please specify)
- Don't know/unsure

Q11b

[Ask if "Prior therapy not having an adequate effect on symptom reduction" was selected for Q9a (change to current treatment regimen)]

You mentioned that inadequate symptom control played a role in changing treatment from [_] to [_] (current regimen). Which particular symptoms were not adequately controlled and led to the requirement for treatment change? Please select all that apply.

Positive symptoms

- Delusions
- Conceptual disorganization
- Hallucinatory behavior
- Excitement/agitation
- Hostility/aggression

Negative symptoms

- Blunted affect
- Emotional withdrawal
- Poor rapport
- Social withdrawal

Cognitive symptoms

- Cognitive dysfunction

Other

- Other symptoms (please specify)
- Don't know/unsure

Q11bi

How would you rate this patient's positive symptomology at the time of treatment change?

[Select one, if applicable]

- Minimal – questionable, subtle or suspected pathology

- Mild – symptom’s presence clearly established but not pronounced
- Moderate – symptom represents serious problem but occurs occasionally or only intrudes on daily life to a moderate extent
- Moderate severe – manifestations that distinctly impact on functioning but are not ‘all-consuming’ and can be contained at will
- Severe – symptom presents frequently, is highly disruptive to patient’s life, and calls for direct supervision
- Extreme – most serious level of psychopathology where manifestations drastically interfere with life functions and close supervision is required

Q11bii

How would you rate this patient’s negative symptomology at the time of treatment change?

[Select one, if applicable]

- Minimal – questionable, subtle or suspected pathology
- Mild – symptom’s presence clearly established but not pronounced
- Moderate – symptom represents serious problem but occurs occasionally or only intrudes on daily life to a moderate extent
- Moderate severe – manifestations that distinctly impact on functioning but are not ‘all-consuming’ and can be contained at will
- Severe – symptom presents frequently, is highly disruptive to patient’s life, and calls for direct supervision
- Extreme – most serious level of psychopathology where manifestations drastically interfere with life functions and close supervision is required

Q11biii

How would you rate this patient’s cognitive symptomology at the time of treatment change?

[Select one, if applicable]

- Minimal – questionable, subtle or suspected pathology
- Mild – symptom’s presence clearly established but not pronounced
- Moderate – symptom represents serious problem but occurs occasionally or only intrudes on daily life to a moderate extent
- Moderate severe – manifestations that distinctly impact on functioning but are not ‘all-consuming’ and can be contained at will
- Severe – symptom presents frequently, is highly disruptive to patient’s life, and calls for direct supervision
- Extreme – most serious level of psychopathology where manifestations drastically interfere with life functions and close supervision is required

Q11c

[Ask if “Prior therapy not having an adequate effect on symptom reduction” was selected for Q9a (change to current treatment regimen)]

How would you describe the level of severity of this patient’s symptoms on the following domains at the time of treatment change from [_] to [_] (current regimen)?

Q11ci Impacted socially useful activities, including work and study

[Select one]

- Absent
- Mild
- Manifest
- Marked
- Severe
- Very severe
- Don’t know

Q11cii Impacted personal and social relationships

[Select one]

- Absent
- Mild
- Manifest
- Marked
- Severe
- Very severe
- Don’t know

Q11ciii Impacted self-care

[Select one]

- Absent
- Mild
- Manifest
- Marked
- Severe
- Very severe
- Don’t know

Q11civ Impacted disturbing and aggressive behaviors

[Select one]

- Absent
- Mild
- Manifest

- Marked
- Severe
- Very severe
- Don't know

Q12

Which of these adjunct therapies, if any, have you ever prescribed for this patient whilst they have been under your care?

[Select all that apply]

- Mood stabilizer
- Antidepressant
- Anxiolytic medication
- Other [please specify]
- None

Q13

When this patient entered your care, how were you able to ascertain this patient's treatment history? Please select all options that apply.

[Select all that apply]

- By accessing patient's medical records/hospital records
- Through feedback from the patient's family
- Through feedback from patient themselves
- No information available on patient's treatment history
- Other (please specify)

Disease history and symptomology

Q14a

What symptoms of schizophrenia does this patient currently experience (either chronically or as breakthrough symptoms)?

[Select all that apply]

Positive symptoms

- Delusions
- Conceptual disorganization
- Hallucinatory behavior
- Excitement/agitation
- Hostility/aggression

Negative symptoms

- Blunted affect
- Emotional withdrawal
- Poor rapport
- Social withdrawal

Cognitive symptoms

- Cognitive dysfunction

Other

- Other symptoms (please specify)
- None

Q14bi

How would you rate this patient's positive symptomology currently in terms of severity?

[Select one, if applicable]

- Minimal – questionable, subtle or suspected pathology
- Mild – symptom's presence clearly established but not pronounced
- Moderate – symptom represents serious problem but occurs occasionally or only intrudes on daily life to a moderate extent
- Moderate severe – manifestations that distinctly impact on functioning but are not 'all-consuming' and can be contained at will
- Severe – symptom presents frequently, is highly disruptive to patient's life, and calls for direct supervision
- Extreme – most serious level of psychopathology where manifestations drastically interfere with life functions and close supervision is required

Q14bii

How would you rate this patient's negative symptomology currently in terms of severity?

[Select one, if applicable]

- Minimal – questionable, subtle or suspected pathology
- Mild – symptom's presence clearly established but not pronounced
- Moderate – symptom represents serious problem but occurs occasionally or only intrudes on daily life to a moderate extent
- Moderate severe – manifestations that distinctly impact on functioning but are not 'all-consuming' and can be contained at will
- Severe – symptom presents frequently, is highly disruptive to patient's life, and calls for direct supervision
- Extreme – most serious level of psychopathology where manifestations drastically interfere with life functions and close supervision is required

Q14biii

How would you rate this patient's cognitive symptomology currently in terms of severity?

[Select one, if applicable]

- Minimal – questionable, subtle or suspected pathology
- Mild – symptom's presence clearly established but not pronounced
- Moderate – symptom represents serious problem but occurs occasionally or only intrudes on daily life to a moderate extent
- Moderate severe – manifestations that distinctly impact on functioning but are not 'all-consuming' and can be contained at will
- Severe – symptom presents frequently, is highly disruptive to patient's life, and calls for direct supervision
- Extreme – most serious level of psychopathology where manifestations drastically interfere with life functions and close supervision is required

Q15

How would you describe the level of severity of these current symptoms on the following domains?

Q15i Impacted socially useful activities, including work and study

[Select one]

- Absent
- Mild
- Moderate
- Marked
- Severe
- Very severe

Q15ii Impacted personal and social relationships

[Select one]

- Absent
- Mild
- Moderate
- Marked
- Severe
- Very severe

Q15iii Impacted self-care

[Select one]

- Absent
- Mild
- Moderate

- Marked
- Severe
- Very severe

Q15iv Disturbing and aggressive behaviors

[Select one]

- Absent
- Mild
- Moderate
- Marked
- Severe
- Very severe

Q16a

Of the positive symptoms of schizophrenia that this patient currently experiences, which symptom do you think is most important to eliminate, in order to improve their long-term prognosis?

Please select the ONE symptom that you believe will have the greatest impact in the long term for this patient.

[Select one from Q14a answer]

Q16b

Of the negative symptoms of schizophrenia that this patient currently experiences, which symptom do you think is most important to eliminate, in order to improve their long-term prognosis?

Please select the ONE symptom that you believe will have the greatest impact in the long term for this patient.

[Select one from Q14a answer]

Q17

[Ask if any positive/negative/cognitive symptom was selected in Q14a]

Approximately how often does the patient experience severe and significantly impairing symptoms of schizophrenia?

	Positive symptoms	Negative symptoms	Cognitive symptoms
A few times a year			
Once every few months			
Around once a month			

3–4 times a month			
2–3 times a week			
On a daily basis			

[Select one for each column]

Q18a

What, if any, comorbid psychiatric conditions or issues has the patient experienced at any point since their diagnosis with schizophrenia?

[Select all that apply]

- Beliefs about bodily illness or malfunctions
- Anxiety
- Feelings of guilt
- Unusual mannerisms or posturing
- Depression
- Cognitive dysfunction (e.g., poor executive functioning, problems focusing, poor working memory)
- Poor impulse control
- Obsessions/compulsions
- Substance abuse/dependence
- Suicidal ideation
- Insomnia
- Any other comorbid psychiatric condition(s) (please specify)
- None
- Don’t know/unsure

Q18b

What, if any, physical comorbidities/risk factors or issues has the patient experienced at any point since their diagnosis with schizophrenia?

[Select all that apply]

- Diabetes
- Hypertension
- Obesity
- Cardiovascular disorders
- Dyslipidemia
- Metabolic syndrome
- Smoking
- Thyroid dysfunction

- COPD/asthma
- Sleep apnea
- Chronic pain
- Cancer
- Any other physical health condition (please specify)
- None
- Don't know/unsure

Q19

How satisfied would you say you are with this patient's progress since diagnosis, in terms of overall improvement in symptomology, behavior, and quality of life?

[Select on scale from 1=not at all satisfied to 10=extremely satisfied]

Q20a

[Ask if TRS]

Thinking about the classification of this patient as having TRS: to the best of your knowledge, after how many failures of different antipsychotic medication trials was it decided that this patient was resistant to treatment? Please include all trials, including failure due to non-adherence, tolerability issues, inadequate dosing, inadequate duration, concurrent illicit substance use, etc.

[Type answer or select 'Don't know/unsure']

__ antipsychotic therapy trials

Q20b

[Ask if TRS]

To the best of your knowledge, after how many failures of different adequate antipsychotic medication trials was it decided that this patient was resistant to treatment? In this context, adequate trials means: "prescription for adequate duration and dose; with adequate affirmation of treatment adherence; and abstinence from CNS-active illicit drugs".

[Type answer or select 'Don't know/unsure']

__ fair antipsychotic therapy trials

Q21a

[Ask if TRS and patient was not prescribed clozapine according to Q7a]

According to your earlier answer, this patient has not received clozapine. Which of the following, if any, apply to this patient?

[Select all that apply]

- Patient is not eligible for clozapine for clinical reasons (i.e., comorbidities)

- Patient is not eligible for clozapine for social/practical reasons
- Patient is not eligible for clozapine because I have concerns about their ability to adhere to blood monitoring requirements
- I have recommended clozapine to this patient in the past but they have refused it
- Patient has not taken clozapine for another reason (please specify)
- None of the above

Q21b

[Ask if TRS and patient was prescribed clozapine according to Q7a]

According to your earlier answer, this patient has received clozapine. Which of the following, if any, apply to this patient?

[Select all that apply]

- Patient requested clozapine
- Patient’s family or significant other requested clozapine
- All other drug therapy options were excluded/unsuitable
- Patient has used/is using clozapine for another reason (please specify)
- None of the above

[Repeat Q1–Q21 for a total of three loops, covering three patients]

Section 2: Drivers, barriers and behaviors

Thank you for providing this information.

Q22a

Considering the [_] patients within your care who have treatment-resistant schizophrenia (TRS): what proportion (%) fall into the following categories regarding the number of antipsychotic agents currently being used to treat their schizophrenia?

Please consider only the typical/atypical antipsychotic agents in use, disregarding anxiolytics or other supportive medication.

[Type answer for each item]

- Treated with monotherapy (one antipsychotic agent)
- Treated with polypharmacy/combination treatment (more than one antipsychotic agent)
- Other approach (please specify)

Q22b

Considering the total population of patients with TRS within your care who are being treated with monotherapy: what proportion (%) fall into the following categories regarding the type of antipsychotic agents currently being used to treat their schizophrenia?

Please consider only the typical/atypical antipsychotic agents in use, disregarding anxiolytics or other supportive medication.

[Type answer for each item]

- Treated with one typical antipsychotic agent
- Treated with one atypical antipsychotic agent
- Other approach (please specify)

Q22c

Considering the total population of patients with TRS within your care who are being treated with antipsychotic polypharmacy/combination treatment: what proportion (%) fall into the following categories regarding the type of antipsychotic agents currently being used to treat their schizophrenia?

Please consider only the typical/atypical antipsychotic agents in use, disregarding anxiolytics or other supportive medication.

LAIs would be included in either typical or atypical depending on the type.

[Type answer for each item]

- Treated with two or more typical antipsychotic agents (combination therapy)
- Treated with two or more atypical antipsychotic agents (combination therapy)
- Treated with a combination of one or more typical antipsychotic agent plus one or more atypical antipsychotic agent (combination therapy)

- Other approach (please specify)

Q23

Considering patients with TRS specifically: from the list of products below, rank your top five antipsychotics in terms of your overall satisfaction with them, in the context of treating/managing TRS.

Please select the relevant antipsychotics to indicate their ranking within your top five. Indicate an antipsychotic polypharmacy/combination regimen by selecting the relevant antipsychotic molecules.

[From list of options in SQ10, select top 1–5 in order]

Q24

If you are faced with a patient with schizophrenia who has had two prior failures of antipsychotic medication, in what order would you use each of the following options in order to manage their condition?

Please select the options to indicate your preference. If you would not use any of these options, please choose the "would not consider this option" column.

[From list of options below, select 1–10 in order]

- Increase dose of current antipsychotic medication (if tolerated)
- Add a second antipsychotic medication
- Add a third antipsychotic medication
- Add a mood stabilizer
- Add an antidepressant
- Add an anxiolytic medication
- Suggest a long-acting-injectable formulation
- Suggest clozapine
- Suggest switching to a (new) atypical antipsychotic (excluding clozapine)
- Suggest switching to a (new) typical antipsychotic

Q25

Thinking generally about the management of schizophrenia, how would you define 'satisfactory improvement'?

[Type answer]

[End of survey – thank and close]
